# Differential Rates of Perinatal Maturation of Human Primary and Nonprimary Auditory Cortex

**DOI:** 10.1523/ENEURO.0380-17.2017

**Published:** 2018-01-16

**Authors:** Brian B. Monson, Zach Eaton-Rosen, Kush Kapur, Einat Liebenthal, Abraham Brownell, Christopher D. Smyser, Cynthia E. Rogers, Terrie E. Inder, Simon K. Warfield, Jeffrey J. Neil

**Affiliations:** 1 Department of Pediatric Newborn Medicine, Brigham and Women’s Hospital, Harvard Medical School, Boston, MA 02115; 2 Department of Radiology, Boston Children’s Hospital, Harvard Medical School, Boston, MA 02115; 3 Translational Imaging Group, University College London, London, WC1E 7JE United Kingdom; 4 Department of Neurology, Boston Children’s Hospital, Harvard Medical School, Boston, MA 02115; 5 Department of Psychiatry, Brigham and Women’s Hospital, Harvard Medical School, Boston, MA 02115; 6 Department of Neurology, Washington University School of Medicine, St. Louis, MO 63130; 7 Department of Pediatrics, Washington University School of Medicine, St. Louis, MO 63130; 8 Department of Psychiatry, Washington University School of Medicine, St. Louis, MO 63130

**Keywords:** audition, cortical development, diffusion tensor imaging, neuroimaging, preterm infants

## Abstract

Primary and nonprimary cerebral cortex mature along different timescales; however, the differences between the rates of maturation of primary and nonprimary cortex are unclear. Cortical maturation can be measured through changes in tissue microstructure detectable by diffusion magnetic resonance imaging (MRI). In this study, diffusion tensor imaging (DTI) was used to characterize the maturation of Heschl’s gyrus (HG), which contains both primary auditory cortex (pAC) and nonprimary auditory cortex (nAC), in 90 preterm infants between 26 and 42 weeks postmenstrual age (PMA). The preterm infants were in different acoustical environments during their hospitalization: 46 in open ward beds and 44 in single rooms. A control group consisted of 15 term-born infants. Diffusion parameters revealed that (1) changes in cortical microstructure that accompany cortical maturation had largely already occurred in pAC by 28 weeks PMA, and (2) rapid changes were taking place in nAC between 26 and 42 weeks PMA. At term equivalent PMA, diffusion parameters for auditory cortex were different between preterm infants and term control infants, reflecting either delayed maturation or injury. No effect of room type was observed. For the preterm group, disturbed maturation of nonprimary (but not primary) auditory cortex was associated with poorer language performance at age two years.

## Significance Statement

Different brain regions mature at different rates, particularly early in development. Knowledge of when specific sensory brain regions are maturing is critical for understanding the susceptibility to external sensory influences (e.g., during critical periods) and potential for vulnerability to injury. Here, we demonstrate in vivo that, during the perinatal period, human primary auditory cortex (pAC) matures earlier than nonprimary auditory cortex (nAC), consistent with accounts of brain development from histology. However, we detect more rapid changes in nAC during this period. Our findings indicate that disruption of nonprimary cortex (but not primary cortex) maturation during this developmental period is associated with poorer childhood language development. Differential developmental timelines may render nonprimary sensory cortex more vulnerable than primary sensory cortex.

## Introduction

Humans are altricial mammals with precocial hearing. An abundance of neurobiological evidence clearly demonstrates that the human auditory system comes online at least as early as 25 weeks postmenstrual age (PMA; Graziani et al., 1968; Starr et al., 1977; Birnholz and Benacerraf, 1983; Rotteveel et al., 1987; Hepper and Shahidullah, 1994), some 15 weeks before term birth. By this age, the structural development of the nervous system is sufficient for peripheral auditory input to reach auditory cortex (Weitzman and Graziani, 1968; Rotteveel et al., 1987; Jardri et al., 2008; Mahmoudzadeh et al., 2013). Furthermore, cortical memory traces are forming long before term birth for auditory input arising from acoustic stimuli in the extrauterine environment, including speech and language (DeCasper and Fifer, 1980; Decasper and Spence, 1986; Moon et al., 1993; Mahmoudzadeh et al., 2013; Moon et al., 2013; Partanen et al., 2013). This makes auditory cortex unique among the sensory cortices and therefore of particular interest when evaluating cortical maturation processes in humans.

During development, neuronal genesis and differentiation occur in primary sensory cortical regions before nonprimary and association regions (Conel, 1939; Sidman and Rakic, 1982), indicating that primary sensory cortex matures in advance of nonprimary cortex. However, the differences between the timelines for primary versus nonprimary cortex maturation are unclear. It may be that nonprimary cortex develops with a rate of maturation identical to, but delayed from, that of primary cortex. On the other hand, nonprimary cortex might follow an altogether different rate of maturation from that of primary cortex. This distinction has implications for the timing and severity of disruption and/or injury to developing cortex and the consequences thereof.

Diffusion tensor imaging (DTI) permits tracking of human cortical maturation *in vivo* through the parameters of fractional anisotropy (FA), mean diffusivity (MD), axial diffusivity (AD), and radial diffusivity (RD; McKinstry et al., 2002; Ball et al., 2013; Smyser et al., 2015). FA, which reflects the degree of anisotropy of water molecule displacements in brain tissue, decreases in developing gray matter (GM) as histologic changes disrupt the initial radial organization of the cortical plate (McKinstry et al., 2002). In developing white matter (WM), FA values increase as preoligodendroglial ensheathment and myelination inhibit water displacements orthogonal to maturing axons. MD, AD, and RD, which measure the mean, axial, and radial magnitude of water displacements, respectively, decrease in both maturing GM and WM as brain water content decreases and cell density increases. It has been demonstrated that cortical GM tissue matures in synchrony with underlying subplate and WM (Kostović et al., 2014; Smyser et al., 2015), suggesting that diffusion parameters from both GM and adjacent subplate/WM reflect the maturational status of developing cortex.

**Figure 1.**
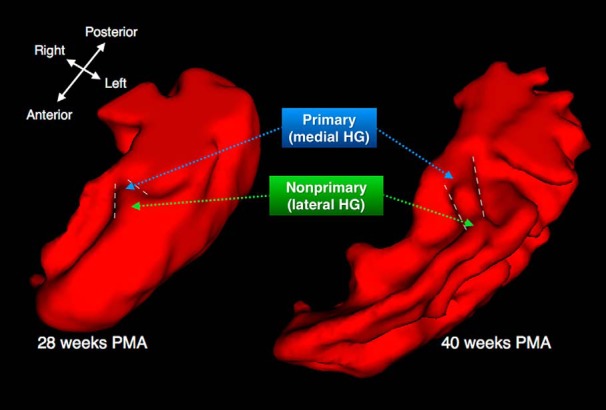
Development of the left hemisphere temporal plane from 28 to 40 weeks PMA. HG (demarcated with white dashed lines) is forming by 28 weeks PMA and has an adult-like appearance by 40 weeks PMA. pAC and nAC are located in medial and lateral HG, respectively.

We used DTI to investigate the maturational timelines of the cortical plate and adjacent subcortical tissue between 26 and 42 weeks PMA in auditory cortex regions in preterm infants. We examined the variation in maturational timeline along the axis of Heschl’s gyrus (HG) in the temporal lobe where cortex transitions from primary auditory cortex (pAC, postero-medially) to nonprimary auditory cortex (nAC, antero-laterally; Fig. 1; Morosan et al., 2001; Moerel et al., 2014). We compared the timeline of maturation of pAC versus nAC, hypothesizing that pAC would mature in advance of nAC. We further hypothesized that different acoustic environments during the perinatal period would affect the maturational timeline of auditory cortex. Finally, we hypothesized that disturbed auditory cortex maturation in infancy would be related to poorer language development in childhood. We tested our hypotheses in a cohort of 90 very preterm infants (born <30 weeks’ gestation) who underwent diffusion magnetic resonance imaging (MRI) up to four times during their hospital stay and neurodevelopmental follow-up at two years old.

## Materials and Methods

### Subjects

**Figure 2.**
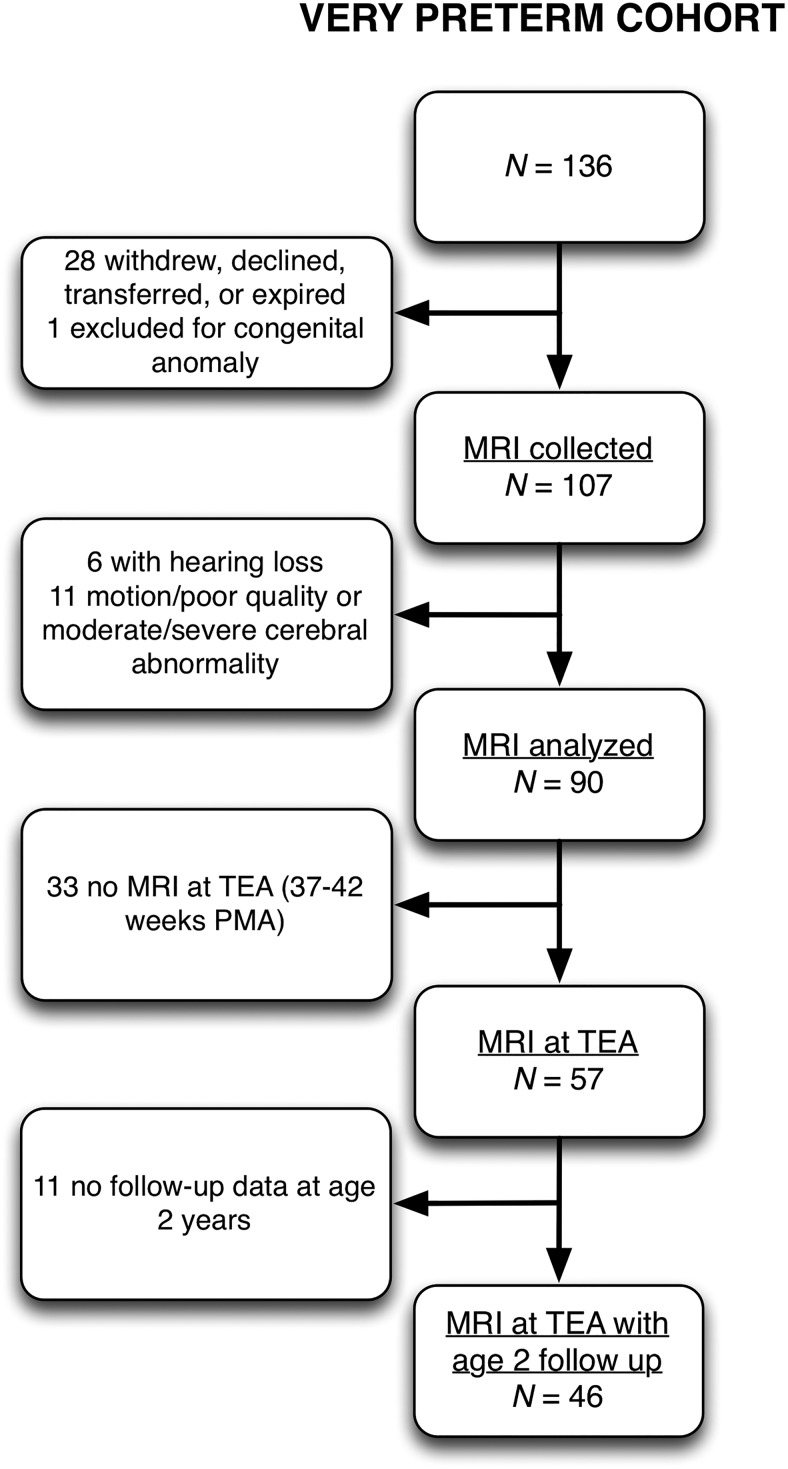
Flowchart detailing retention and follow-up of the very preterm cohort. TEA, term-equivalent age.

A total of 136 very preterm infants born before 30 weeks’ gestation were recruited from the St. Louis Children’s Hospital Neonatal Intensive Care Unit (NICU) between 2007 and 2010. Infants with moderate to severe (Kidokoro et al., 2013) WM injury or severe brain abnormalities were excluded from the analysis (Fig. 2). Infants with any conductive or sensorineural hearing loss (assessed after discharge for infants who failed newborn hearing screening) were likewise excluded. Infants who failed newborn hearing screening but underwent no audiological follow-up were also excluded. Infants underwent MRI one to four times during hospital stay based on their clinical stability to travel to the MRI scanner. Of those meeting selection criteria, 90 subjects had usable MRI data collected at some point during hospital stay, with 56 subjects imaged at multiple timepoints, for a total of 173 images between 26 and 42 weeks PMA. Of the 90 subjects, 57 had data collected at term-equivalent age (37–42 weeks PMA). Infants in the NICU environment were pseudo-randomly assigned to either a noisier open bay multi-bed unit (*N* = 46) or a quieter single patient room (*N* = 44) based on staffing and bed availability (according to standard clinical practice), but otherwise had access to the same medical care and physicians, as described elsewhere (Pineda et al., 2014). Fifteen healthy term-born control infants were recruited from the Barnes-Jewish Hospital Newborn Nursery and scanned within the first 4 d of life. Term infants had no history of illicit substance exposure *in utero* and no evidence of acidosis in the first hour of life. No infants had chromosomal abnormalities or congenital infections. Informed written parental consent was obtained for each subject. The study was approved by the Washington University Human Studies Committee.

Participants in the preterm group returned for follow-up assessment at two years old and were assessed with the Bayley Scales of Infant and Toddler Development, 3rd edition (Bayley and Reuner, 2006). Outcomes used for this study were scores from the receptive communication subtest (assessing preverbal behaviors, verbal comprehension and vocabulary), expressive communication subtest (assessing preverbal babbling and gesturing, as well as vocabulary and utterances), and cognitive subtest (assessing sensorimotor development, memory, object relatedness, and concept formation). Of those with useable MRI data at term-equivalent age (*N* = 57), 46 had behavioral data collected at two years old (for characteristics of these infants, see Table 1). The 11 infants who were not assessed at two years old had a higher average birth weight (1158 vs 935 g, *p* = 0.03) and a lower average maternal age (24 vs 29 years, *p* = 0.04) than the other 46 infants, but otherwise did not have significantly different characteristics.

**Table 1.** Sample characteristics for children followed up at two years old, separated by room type

	Open bay (*n* = 22)	Private room (*n* = 24)	*p* value
	*Mean (SD)*		
Birth GA (weeks)	26.3 (1.7)	27 (2.1)	0.20
Birthweight (grams)	883 (242)	983 (250)	0.17
Length of stay (days)	92 (18)	91 (25)	0.81
PMA at scan (weeks)	37.8 (1.2)	38.3 (1.5)	0.16
Maternal age (years)	29.1 (8.5)	29.8 (7.3)	0.79
Social risk score (out of 5)	1.45 (1.34)	1.30 (1.26)	0.70
	*n* (%)		
Male	10 (45)	10 (42)	0.80
SGA	1 (5)	1 (4)	0.95
Infection	4 (18)	2 (9)	0.38
BPD	12 (55)	15 (68)	0.35

GA, gestational age; SGA, small for gestational age; BPD, bronchopulmonary dysplasia.

### MRI data acquisition and processing

Infants were imaged during natural sleep or quiescence without the use of sedation. Infants wore neonatal earmuffs (Natus Medical) for hearing protection. Heart rate and arterial oxygen saturation were monitored continuously throughout data acquisition. Diffusion MRI data were acquired with a single-shot echo-planar sequence (repetition time/echo time 13,300/112 ms, 1266 Hz/Px bandwidth, 128-mm field of view, voxel size 1.2 × 1.2 × 1.2 mm^3^, 48 *b*-directions with multiple amplitudes ranging from 0 to 1200s/mm^2^) using a 3-T Siemens TIM Trio system with an infant-specific quadrature head coil (Advanced Imaging Research). Other MRI data collected included: rapid gradient echo T1-weighted images (repetition time/echo time 1500/3 ms, voxel size 1 × 0.7 × 1 mm^3^) and fast spin echo T2-weighted images (repetition time/echo time 8500/160 ms, voxel size 1 × 1 × 1 mm^3^). Total data acquisition time was ∼60 min.

MD, AD, RD, and FA values were estimated using a weighted linear least square approach, implemented in FSL v5.0.2 (Jenkinson et al., 2012). The FA noise floor was ∼0.06. (To determine FA noise floor, we randomly selected three infants and sampled a region of cerebrospinal fluid, which is known to have low FA. Each sample consisted of ≥60 contiguous voxels. The average FA for these samples was 0.062, 0.058, and 0.044.)

Regions of interest (ROIs) were placed manually by an expert rater in native space using T2-weighted (b = 0 s/mm^2^) images and MD parametric maps to identify the cortical GM and adjacent WM of Heschl’s gyrus (HG) in the left hemisphere. HG was defined using a previously established method based on anatomic landmarks (Penhune et al., 1996). In cases of HG duplications, only the anterior gyrus was used for analysis. The boundary between cortical GM and WM was determined by using an intensity threshold value based on histograms generated from initial manual selection of low-intensity voxels reliably identified as HG cortical tissue and high-intensity voxels reliably identified as WM. To minimize partial volume effects, any voxels whose intensity values were ambiguous (i.e., were between threshold values for GM and WM), were excluded from analysis. ROIs were subdivided into single-voxel-thick oblique “slices” in equidistant steps along the length of HG. Average FA, MD, AD, and RD were obtained for each tissue type in each ROI slice. On average, the number of slices required to define the length of HG was 13 slices for the earliest ages (26–28 weeks PMA) and 29 slices at term-equivalent age (37–42 weeks PMA).

For region comparison (primary versus nonprimary), each diffusion parameter was averaged over the first three slices for pAC, beginning with the most postero-medial slice and moving laterally. The use of three slices ensured that tissues were well within the putative boundary between pAC (areas Te1.0/1.1) and nAC (area Te1.2), believed to be one-half to two-thirds the length of HG (Rademacher et al., 1993; Penhune et al., 1996; Morosan et al., 2001; Glasser and Van Essen, 2011; Clarke and Morosan, 2012; Marie et al., 2015). For nAC, each parameter was averaged over the most lateral three slices. The intra-rater reliability rating for pAC FA was 0.83, while reliability for all other regions and tissues was >0.88. For group comparisons at term-equivalent age, ROIs were also generated for whole-brain cortical GM and cerebral WM using a preterm-specific automated segmentation algorithm (Cardoso et al., 2013), using tissue class priors from a T2-weighted longitudinal atlas (Kuklisova-Murgasova et al., 2011).

**Figure 3.**
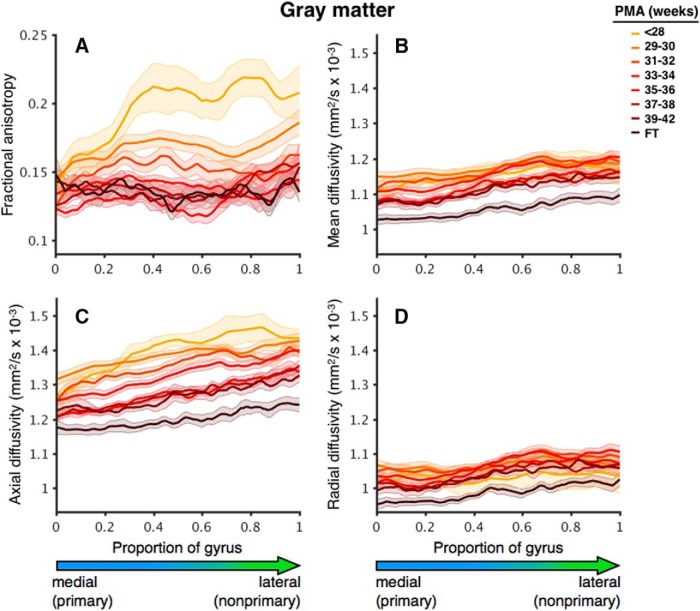
Changes in GM diffusion parameters with increasing age along the length of HG from medial (primary) to lateral (nonprimary). Changes are shown for (***A***) FA, (***B***) MD, (***C***) AD, and (***D***) RD. Curves are separated by age group (in weeks), indicated by color. Shading indicates ±1 SE. FT, full-term controls.

For data visualization in Figures 3, 5, slices were interpolated to 100 points along the length of HG for each dataset. Datasets were then binned into groups based on PMA in weeks [≤28 (*N* = 7), 29–30 (*N* = 25), 31–32 (*N* = 20), 33–34 (*N* = 44), 35–36 (*N* = 20), 37–38 (*N* = 42), and 39–42 (*N* = 15)] and averaged.

### Experimental design and statistical analysis

Changes in diffusion parameters over time were modeled as 3-level linear mixed-effects models which allowed for nesting of repeated observations within regions and regions within subjects. The model for the mean response included the main effect of region indicator variable, and linear and quadratic trends of age along with their interactions with the region indicator variable as the fixed effects. In addition, it included random effects of intercept and slope to account for correlations within regions and within time points nested within regions. The final model selection was performed using Akaike Information criterion (AIC). Likelihood ratio test (LRT) for the nested models was also used to test the significance of variance covariance parameters of the random effects. A reduced 2-level mixed-effects model was fit in cases where the variance components were not found to significantly explain the correlations within regions and/or within time points within regions.

Group (preterm versus full term) and region (primary versus nonprimary) differences in FA, MD, AD, and RD for each tissue type were assessed at term-equivalent age (37–42 weeks PMA) using a single, 2-level linear mixed-effects model for each parameter and tissue type. Each model included a group × region interaction term and controlled for PMA at the time of scan. Interaction terms were removed from the model if found to be not significant. To determine whether observed differences were specific to auditory cortex, a second model for each analysis controlled for the corresponding whole-brain diffusion parameter values.

Associations between language outcomes (receptive language and expressive language) at two years old and diffusion parameters at term-equivalent age were tested for the preterm group using separate linear regression models for each parameter (FA, MD, AD, and RD), tissue type (GM and WM), and region (primary and nonprimary). Each model controlled for room type since room type was known to affect language outcome in these patients (Pineda et al., 2014). Models in a secondary analysis also controlled for birth gestational age and a social risk score based on a 5-point scale, calculated as the sum of binary values indicating the presence or absence of (1) maternal education level lower than high school diploma, (2) African-American race, (3) public insurance, (4) maternal age <19 years, and (5) single-parent household. For each outcome and tissue type, significance values were adjusted for multiple comparisons using Bonferroni correction. As additional controls, we also tested for associations (1) between whole-brain diffusion parameters at term-equivalent age and language outcomes at two years old, and (2) between auditory cortex parameters at term-equivalent age and cognitive composite scores at two years old. Analyses for term-equivalent age data were performed using R (R Core Team, 2016). All other analyses were performed using SAS (SAS Institute Inc.).

## Results

### Cortical gray matter

**Figure 4.**
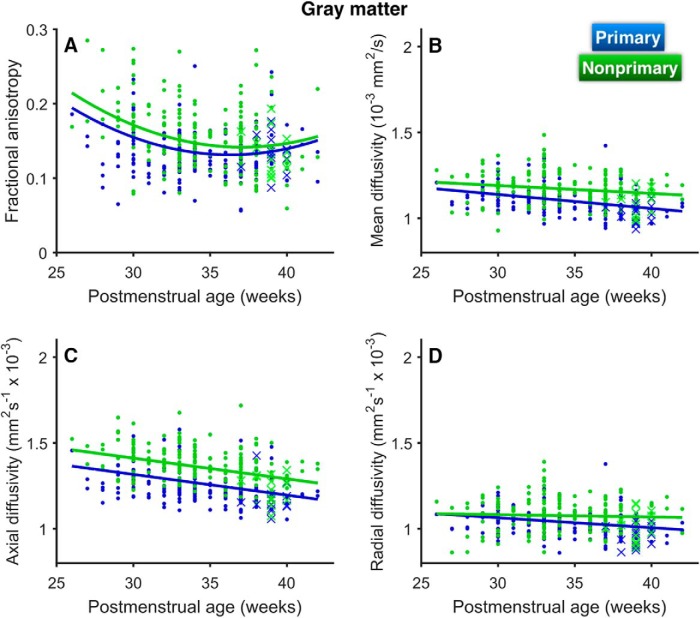
Changes in GM diffusion parameters as a function of PMA for primary (blue) and nonprimary (green) auditory cortex in HG. Changes are shown for (***A***) FA, (***B***) MD, (***C***) AD, and (***D***) RD. Curves represent results from linear mixed-effects models. Values for full-term controls are denoted by crosses (×).

At 28 weeks PMA, FA was lower in pAC and higher in nAC, as can be seen in the plot corresponding to <28 weeks PMA in Figure 3*A*. Note that the FA values are higher in the lateral (nonprimary) portion of the gyrus. FA decreased with increasing PMA with a quadratic trend (Table 2; Fig. 4*A*). FA decreased more sharply for nAC than for pAC. As a result, FA values for nAC, while significantly higher than pAC values at 30 (*p* = 0.001) and 35 weeks PMA (*p* < 0.001), reached similar values by 40 weeks PMA (*p* = 0.24). An effect of cortical region (primary versus nonprimary) was observed, with lower FA in pAC and no significant interaction between region and age.

**Table 2.** Regression coefficients showing effects of age and region on diffusion parameter changes

		Age^2^		Age		Region		Age × region	
		Coefficient	*p* value	Coefficient	*p* value	Coefficient	*p* value	Coefficient	*p* value
GM	FA	0.594	<0.001	−13.163	<0.001	−20.076	**0.013**	0.926	0.304
	MD	—	—	−4.603	0.001	−37.863	**0.028**	−3.533	0.068
	AD	—	—	−12.055	<0.001	−94.541	**<0.001**	−1.729	0.417
	RD	—	—	−1.333	0.388	−16.440	0.379	−4.457	**0.035**
WM	FA	—	—	3.096	<0.001	24.853	**<0.001**	−1.481	**0.036**
	MD	−0.976	0.006	−10.189	0.081	−189.456	**<0.001**	7.976	**0.001**
	AD	−0.781	0.044	−11.068	0.092	−164.175	**<0.001**	5.683	**0.033**
	RD	−1.060	0.003	−9.959	0.088	−202.240	**<0.001**	9.130	**<0.001**

Bold values indicate significant effects of region and age × region interactions. Coefficient values are multiplied by 10^3^.

Both MD and AD decreased with increasing PMA, with effects of region (lower in pAC) and no interactions between region and age (Figs. 3*B*,*C*, 4*B*,*C*; Table 2). There was a region × age interaction for the decreases in GM RD, with pAC RD declining significantly with age (*p* < 0.001) and nAC RD showing no linear trend (*p* = 0.39; Figs. 3*D*, 4*D*).

The group comparison at term-equivalent age revealed that preterm infants had higher values for GM MD (*p* < 0.001), AD (*p* < 0.001), and RD (*p* = 0.004) than healthy full-term infants (Fig. 3). No effect of prematurity was observed in FA (*p* = 0.69). An effect of cortical region was observed for FA (*p* = 0.04), MD (*p* < 0.001), AD (*p* < 0.001), and RD (*p* < 0.001), with nAC values higher than pAC values. No interactions between group and cortical region were observed for GM diffusion measures. The group difference in AD remained significant when controlling for whole-brain GM values (*p* = 0.04), while group differences in all other parameters were diminished.

For the preterm group, we found no effect of room environment (open bay versus single patient room) on any GM diffusion measures. For the preterm group, higher FA in nAC at term-equivalent age was associated with poorer expressive language performance at two years old (Tables 3, 4). pAC and whole-brain cortical GM FA showed no such association with expressive language performance (corrected *p* > 0.9). No associations were observed between GM diffusion parameters and receptive language. (Two associations observed between nAC diffusion parameters and receptive language did not remain significant after correction for multiple comparisons.) No associations were observed between auditory GM diffusion parameters and cognitive composite score.

**Table 3.** Regression coefficients showing associations between diffusion parameter values at term-equivalent age and language ability at two years old, controlling for room type

		Receptive			Expressive		
		Coefficient	Corrected *p* value	*R*^2^	Coefficient	Corrected *p* value	*R*^2^
pGM	FA	−7.08	>0.99	0.04	10.83	>0.99	0.09
	MD	9.43	>0.99	0.07	9.07	>0.99	0.10
	AD	6.26	>0.99	0.05	9.91	>0.99	0.12
	RD	9.10	>0.99	0.07	6.51	>0.99	0.09
nGM	FA	−10.46	>0.99	0.06	−29.39	**0.04**	0.23
	MD	12.96	0.10	0.16	10.41	0.68	0.14
	AD	8.69	0.33	0.12	1.63	>0.99	0.08
	RD	11.74	0.14	0.15	13.12	0.17	0.18
pWM	FA	14.06	>0.99	0.06	−2.71	>0.99	0.08
	MD	2.97	>0.99	0.04	9.63	>0.99	0.12
	AD	5.19	>0.99	0.06	7.96	>0.99	0.12
	RD	0.44	>0.99	0.03	7.87	>0.99	0.11
nWM	FA	−12.37	>0.99	0.05	−14.84	>0.99	0.10
	MD	0.26	>0.99	0.03	3.86	>0.99	0.09
	AD	−0.30	>0.99	0.03	2.27	>0.99	0.08
	RD	0.57	>0.99	0.03	4.38	>0.99	0.10

Bold values indicate a significant association. Coefficient values for diffusivity measures are multiplied by 10^3^. p/nGM, primary/nonprimary GM.

**Table 4.** Regression coefficients showing associations between diffusion parameter values at term-equivalent age and language ability at two years old, controlling for room type, social risk, and birth gestational age

		Receptive			Expressive		
		Coefficient	Corrected *p* value	*R*^2^	Coefficient	Corrected *p* value	*R*^2^
pGM	FA	−0.83	>0.99	0.08	8.66	>0.99	0.09
	MD	9.11	>0.99	0.12	9.07	>0.99	0.11
	AD	7.41	>0.99	0.12	9.55	>0.99	0.12
	RD	8.08	>0.99	0.12	6.85	>0.99	0.10
nGM	FA	−9.10	>0.99	0.10	−32.59	**0.03**	0.26
	MD	12.11	0.22	0.19	12.85	0.37	0.17
	AD	8.05	0.55	0.16	2.62	>0.99	0.09
	RD	11.15	0.30	0.18	16.23	0.07	0.23
pWM	FA	15.74	>0.99	0.12	−3.71	>0.99	0.08
	MD	1.78	>0.99	0.09	9.53	>0.99	0.13
	AD	4.39	>0.99	0.10	7.80	>0.99	0.12
	RD	−0.50	>0.99	0.08	7.98	>0.99	0.11
nWM	FA	−8.82	>0.99	0.09	−19.28	>0.99	0.11
	MD	−0.06	>0.99	0.08	5.11	>0.99	0.11
	AD	−0.47	>0.99	0.09	3.10	>0.99	0.09
	RD	0.20	>0.99	0.08	5.68	>0.99	0.12

Bold values indicate a significant association. Coefficient values for diffusivity measures are multiplied by 10^3^. p/nGM, primary/nonprimary GM.

### White matter

**Figure 5.**
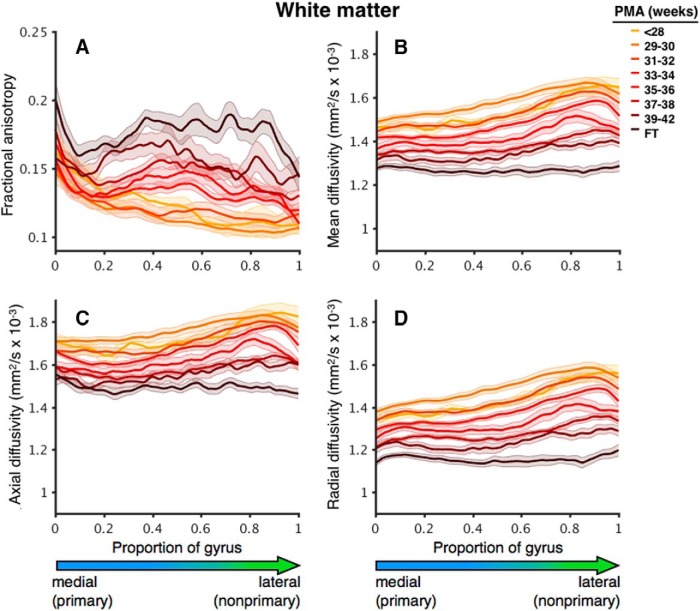
Changes in WM diffusion parameters with increasing age along the length of HG from medial (primary) to lateral (nonprimary). Changes are shown for (***A***) FA, (***B***) MD, (***C***) AD, and (***D***) RD. Curves are separated by age group (in weeks), indicated by color. Shading indicates ±1 SE. FT, full-term controls.

**Figure 6.**
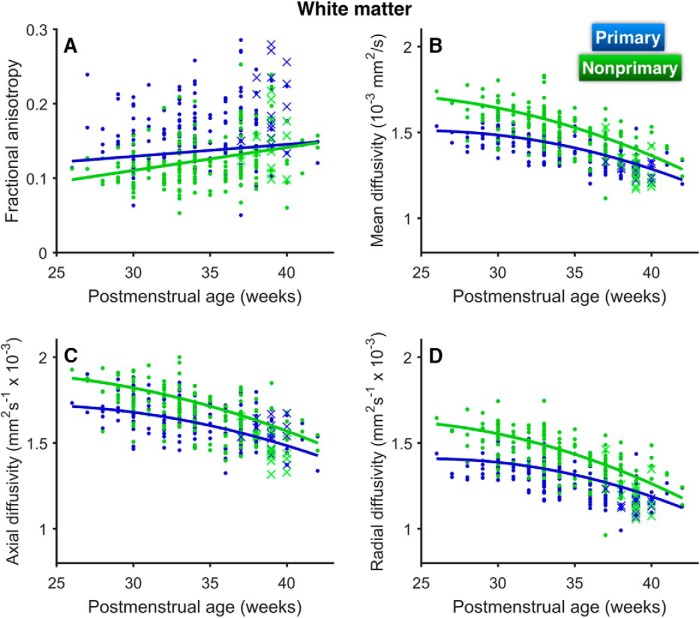
Changes in WM diffusion parameters as a function of PMA for primary (blue) and nonprimary (green) auditory cortex in HG. Changes are shown for (***A***) FA, (***B***) MD, (***C***) AD, and (***D***) RD. Curves represent results from linear mixed-effects models. Values for full-term controls are denoted by crosses (×).

FA for HG WM increased with increasing PMA for the entire length of HG (Table 2; Figs. 5*A*, 6*A*). While an effect of region was observed, with primary WM (pWM) FA values higher than nonprimary WM (nWM) values, more rapid changes were apparent once again in the lateral two-thirds of HG, confirmed by a significant region × age interaction (Table 2). FA values for the middle portion of HG (from 35% to 90% of full HG length), while initially lower than values for the medial region, ultimately reached values similar to that of the most medial point at term-equivalent age (Fig. 5*A*).

WM MD, AD, and RD exhibited similar behavior to one another (Fig. 5*B–D*). Each decreased with age with a significant quadratic trend and an effect of region, with lower values for PWM (Fig. 6*B–D*; Table 2). Each showed a more rapid decline in nWM than in pWM, confirmed by significant region × age interactions (Table 2). Each also showed more dramatic changes than those observed for their GM counterparts.

At term-equivalent age, preterm infants had higher values than healthy full-term infants for WM MD (*p* = 0.004) and RD (*p* = 0.001), whereas higher values for AD (*p* = 0.13) and lower values for FA (*p* = 0.07) did not reach significance (Fig. 5). However, significant interactions between group and cortical region were observed for FA (*p* = 0.02), MD (*p* < 0.001), AD (*p* = 0.005), and RD (*p* < 0.001). In each case, posthoc comparisons showed that the deviation of preterm infants from full-term infants was larger and highly significant in nWM (*p* < 0.001 for all parameters). This analysis also revealed that, for the preterm group, (1) nWM MD, AD, and RD values were significantly higher than those for pWM (*p* < 0.001 for all parameters), and (2) nWM FA was significantly lower than pWM FA (*p* = 0.02). Main effects of group were diminished when controlling for whole-brain values, but all interactions remained significant. We found no effect of room environment on any WM diffusion parameters and no associations between WM diffusion parameters and language outcomes at two years old for the preterm group (Tables 3, 4).

## Discussion

### Cortical gray matter

The asymptotic decline of FA in maturing GM during the perinatal period has been reported previously (McKinstry et al., 2002; Ball et al., 2013; Smyser et al., 2015). Our results indicate that pAC FA reaches a mature value earlier than that observed for the rest of the temporal lobe, prefrontal areas, and other cortical regions (Ball et al., 2013; Smyser et al., 2015), indicating that pAC matures relatively early in development. This observation is in accordance with non-human primate diffusion data (Kroenke et al., 2007).

The neuroanatomical bases for changes to FA and diffusivity measures remain a matter of investigation, but it is likely that decreasing GM FA at this stage reflects a variety of processes – myelination of intra-cortical WM, outgrowth of basal dendrites from pyramidal cells, regression of radial glia, and maturation of interneurons – all of which disrupt the initial radial organization of the maturing cortical plate (McKinstry et al., 2002). The sharper decline in AD and shallower decline in RD observed for GM is consistent with this interpretation, as most of the processes listed above, with the possible exception of regression of radial glia, would hinder water displacements in a radial orientation and thereby reduce AD. Our data suggest that the microstructural maturational processes leading to the decline of FA of the developing cortical plate are established to a considerable degree in pAC by 28 weeks PMA. As a result, the decline in FA we detect in pAC after 28 weeks PMA is relatively modest. In contrast, these processes in nAC are in early stages at 28 weeks PMA, and thus we detect a larger decline in FA.

### White matter

The perinatal period before term is a time of transition for tissue adjacent to the cortical plate. During this time, the transient subplate zone, the largest compartment of the human neocortical wall, involutes and is gradually replaced by developing WM. In humans, the width of the subplate zone is at its maximum around 22–24 weeks PMA, but shows region dependence: the maximum width is ∼1 mm in primary visual cortex and can be nearly 5 mm in somatosensory cortex (Kostović and Rakic, 1990). After 24 weeks PMA, the subplate zone width gradually decreases, initially showing marked thinning adjacent to the depths of the sulci, with a thicker band of subplate neurons observed adjacent to the crowns of the gyri (Kostović et al., 2014). We know of no study that has examined the width of the subplate in pAC in humans, but, given our spatial resolution (1.2 mm), it is likely that our WM analysis captures the changes in diffusion parameters associated with the transition of adjacent tissue from the subplate zone at the earliest time points (26–27 weeks PMA) to maturing WM at the later time points.

We observed region-dependent differences in WM diffusion parameters, with lower FA and higher MD, AD, and RD for nWM. These findings all point to less mature tissue in nWM. At the same time, we also observed that the rates of increase in FA and decrease in MD, AD, and RD were greater for nWM, suggesting that maturing nWM is changing more rapidly than pWM during the perinatal period. We questioned whether the more rapid changes observed in nWM might be a consequence of preterm birth and postnatal events. However, lower FA and higher MD/AD/RD values for nWM (relative to pWM) were observed at our earliest time points (<28 weeks), very shortly after birth for most of these infants. Since developmental changes in diffusion parameters occur on a relatively slow timescale, it is likely that these early diffusion values reflect those that would be observed *in utero*.

Finally, there has been some dispute regarding the topographical boundaries of pAC and nAC in HG, including the definition of a medial/lateral border (Da Costa et al., 2011; Clarke and Morosan, 2012; Moerel et al., 2014). While we are unable to resolve this issue, our data show a clear but gradual transition from pAC to nAC along the axis of HG during development. Our results suggest that the lateral two-thirds of HG follows a maturational process distinct from that of the medial third, whereas the putative boundary between pAC and nAC is believed to be at approximately the medial one-half to two-thirds of HG (Rademacher et al., 1993; Penhune et al., 1996; Clarke and Morosan, 2012; Marie et al., 2015).

### Vulnerability

Maturation of both auditory GM and WM was disrupted by premature birth. The directions of preterm-birth related differences in GM MD/AD/RD and WM FA/MD/AD/RD are all consistent with a delay in maturation and/or injury. Whereas GM appeared equally affected across HG, nWM showed significantly larger deviations from healthy values than did pWM, and we speculate that the primary region is more resistant to disruption from preterm birth. This resistance may be due to the fact that pAC is more mature at the time of preterm birth, but could also be related to the rapidity of tissue changes during the perinatal period. That is, the phenomenon observed here that nWM matures at a more rapid pace (as defined by changes in diffusion measures) than pWM during this period may render nAC/nWM tissue more vulnerable to disruption or injury due to circumstances consequential to preterm birth. Although we observed no regional effects of prematurity in cortical GM, given the importance of the subplate in establishing thalamocortical connections during development (Ghosh et al., 1990; Kanold et al., 2003; Hoerder-Suabedissen and Molnár, 2015), the regional effects we observed in subplate and WM tissue might induce region-specific changes in GM as cortex continues to mature beyond 42 weeks PMA.

Contrary to our hypothesis, microstructural maturation of auditory cortical GM and WM appeared unaffected by acoustic differences between an open-bay NICU and single patient rooms. The open-bay NICU environment tends to be noisier, whereas infants in single patient rooms tend to experience more and longer periods of silence (Jobe, 2014). One recent study suggests infants in single patient rooms experience up to three more hours of silence in a 16-h period than infants in an open-bay NICU, with daily average sounds levels being 2-4 dB higher in the open-bay environment (Pineda et al., 2017). There is some indication that these differences in room environment affect macrostructural development of nonprimary auditory regions (Pineda et al., 2014). Furthermore, animal studies have revealed that sound deprivation (or lack of an enriched auditory environment) can induce deficits in both structure and function of auditory cortex neurons (McMullen and Glaser, 1988; Bose et al., 2010; Mowery et al., 2015). As we detected no microstructural differences associated with the two room types here, it may be that diffusion measures lack sensitivity to these subtler changes in neural structure, or that the acoustic differences between the room types were not sufficiently large to induce such changes. Alternatively, since drastic changes to sensory experience during the perinatal period appear to have a highly selective impact on only some sensory neural development processes, it may be that the differences between acoustic environments produce neural changes undetectable by diffusion MRI. For example, some evidence suggests that altered perinatal sensory experience might induce differences in size and distribution of synapses, whereas synaptogenesis and overall synaptic density is preserved (Bourgeois et al., 1989).

We found that higher FA in nAC at term-equivalent age was associated with poorer expressive communication ability (but not general cognitive functioning) at two years old for preterm infants. Since higher FA can reflect less mature (or injured) GM, this finding suggests that the disruption or delay of maturation of nAC during the perinatal period might have long-lasting consequences. Although we did not observe a strong relationship with receptive communication ability, nAC is intimately involved in the perception of complex sounds including speech and other vocalizations, and therefore important for learning spoken language communication (Davis and Johnsrude, 2003; Liebenthal et al., 2005; Hickok and Poeppel, 2007; Leaver and Rauschecker, 2010; Poeppel, 2014; Norman-Haignere et al., 2015). Additionally, nAC is one of many regions that exhibit functional activity during both speaking and listening (Awad et al., 2007; Stephens et al., 2010; Hagoort, 2014). Thus, it is biologically plausible that a relationship exists between disrupted maturation of nAC and expressive language development. Since human fetuses are learning several aspects of extrauterine vocal communication and language before term (DeCasper and Fifer, 1980; Decasper and Spence, 1986; Moon et al., 1993; Mahmoudzadeh et al., 2013; Moon et al., 2013; Partanen et al., 2013), it is possible that the disrupted maturation of auditory cortex that we observed here could underlie, at least in part, the auditory processing, speech, and language deficits widely reported for preterm infants (Pasman et al., 1992; Davis et al., 2001; Mikkola et al., 2007; Barre et al., 2011; van Noort-van der Spek et al., 2012; Reidy et al., 2013; Vohr, 2014; Paquette et al., 2015). Whereas the auditory periphery and auditory brainstem appear largely unaffected by preterm birth (Eggermont and Salamy, 1988; Jiang, 1995; Eggermont et al., 1996; Tognola et al., 2005; Jedrzejczak et al., 2007; Jiang et al., 2009; Li et al., 2013), our findings suggest that auditory cognitive deficits in preterm infants originate in auditory cortex.

Our data provide *in vivo* demonstration of an important facet of human cortical maturation: primary sensory cortex develops earlier than nonprimary cortex. We were able to distinguish between pAC and nAC as early as 28 weeks PMA, a time at which the sulcal boundaries of HG are just beginning to appear. We found associations between diffusion parameters and preterm birth, and between diffusion FA and language function. In conclusion, diffusion MRI provides a unique window into cortical maturation in human infants, for whom histology is rarely available.

## Synthesis

Reviewing Editor: Margaret McCarthy, Univ of Maryland School of Medicine Decisions are customarily a result of the Reviewing Editor and the peer reviewers coming together and discussing their recommendations until a consensus is reached. When revisions are invited, a fact-based synthesis statement explaining their decision and outlining what is needed to prepare a revision will be listed below. Note: If this manuscript was transferred from JNeurosci and a decision was made to accept the manuscript without peer review, a brief statement to this effect will instead be what is listed below.

This is a revised manuscript transferred from J. Neuroscience. The previous critiques were all related to presentation with no major concerns regarding experimental design or interpretation. There also was no request for additional experiments. The authors have done a thorough job of responding to the previous critiques and have modified the manuscript accordingly, mostly in the realm of additional information on subjects, details on statistical analysis and citations.
